# Pharmacokinetics and effect of intravenous meloxicam in weaned Holstein calves following scoop dehorning without local anesthesia

**DOI:** 10.1186/1746-6148-8-153

**Published:** 2012-09-01

**Authors:** Johann F Coetzee, Ruby A Mosher, Butch KuKanich, Ronette Gehring, Brad Robert, J Brandon Reinbold, Brad J White

**Affiliations:** 1 Department of Clinical Sciences, College of Veterinary Medicine, Kansas State University, 66506-5601, Manhattan, KS, USA; 2 Department of Anatomy and Physiology, Kansas State University, 66506-5601, Manhattan, KS, USA; 3 Present address: Veterinary Diagnostic and Production Animal Medicine, College of Veterinary Medicine, Iowa State University, 50014, Ames, IA, USA

**Keywords:** Analgesia, Meloxicam, Dehorning, Substance P, Cortisol, Heart rate, Accelerometers, Performance

## Abstract

**Background:**

Dehorning is a common practice involving calves on dairy operations in the United States. However, less than 20% of producers report using analgesics or anesthetics during dehorning. Administration of a systemic analgesic drug at the time of dehorning may be attractive to dairy producers since cornual nerve blocks require 10 – 15 min to take effect and only provide pain relief for a few hours. The primary objectives of this trial were to (1) describe the compartmental pharmacokinetics of meloxicam in calves after IV administration at 0.5 mg/kg and (2) to determine the effect of meloxicam (n = 6) or placebo (n = 6) treatment on serum cortisol response, plasma substance P (SP) concentrations, heart rate (HR), activity and weight gain in calves after scoop dehorning and thermocautery without local anesthesia.

**Results:**

Plasma meloxicam concentrations were detectable for 50 h post-administration and fit a 2-compartment model with a rapid distribution phase (mean T_½α_ = 0.22 ± 0.087 h) and a slower elimination phase (mean T_½β_ = 21.86 ± 3.03 h). Dehorning caused a significant increase in serum cortisol concentrations and HR (P < 0.05). HR was significantly lower in the meloxicam-treated calves compared with placebo-treated calves at 8 h (P = 0.039) and 10 h (P = 0.044) after dehorning. Mean plasma SP concentrations were lower in meloxicam treated calves (71.36 ± 20.84 pg/mL) compared with control calves (114.70 ± 20.84 pg/mL) (P = 0.038). Furthermore, the change in plasma SP from baseline was inversely proportional to corresponding plasma meloxicam concentrations (P = 0.008). The effect of dehorning on lying behavior was less significant in meloxicam-treated calves (p = 0.40) compared to the placebo-treated calves (P < 0.01). Calves receiving meloxicam prior to dehorning gained on average 1.05 ± 0.13 kg bodyweight/day over 10 days post-dehorning compared with 0.40 ± 0.25 kg bodyweight/day in the placebo-treated calves (p = 0.042).

**Conclusions:**

To our knowledge, this is the first published report examining the effects of meloxicam without local anesthesia on SP, activity and performance of calves post-dehorning. These findings suggest that administration of meloxicam alone immediately prior to dehorning does not mitigate signs of acute distress but may have long term physiological, behavior and performance effects.

## Background

Dehorning is one of the most common practices involving calves on dairy operations in the United States [1]. Although the American Veterinary Medical Association recommends the use of practices which reduce pain associated with dehorning, there are currently no drugs approved for analgesia in cattle in the United States [2]. Meloxicam is a non-steroidal anti-inflammatory drug (NSAID) of the oxicam class that is approved in the European Union for adjunctive therapy in the treatment of cattle for acute respiratory disease; diarrhea and acute mastitis when administered at 0.5 mg/kg IV or SC [3]. Meloxicam has recently been approved in Canada for improving appetite and weight gains at the onset of diarrhea (calves > 1 w of age) and relief of pain following de-budding of horn buds in calves less than 3 months of age. Heinrich et al. (2009) demonstrated that 0.5 mg/kg meloxicam IM combined with a cornual nerve block reduced serum cortisol response for 6 hours in 6-12 wk old calves compared with calves receiving only local anesthesia prior to cautery dehorning [4]. Furthermore, calves receiving meloxicam had lower heart rates and respiratory rates than placebo treated control calves over 24 hours post-dehorning. Stewart et al. (2009) found that meloxicam administered IV at 0.5 mg/kg mitigated the onset of pain responses associated with hot-iron dehorning in 33 ± 3 day-old calves compared with administration of a cornual nerve block alone as measured by heart rate variability and eye temperature [5]. Heinrich et al. (2010) reported that meloxicam treated calves were less active than controls for 5 hours after dehorning and displayed less sensitivity to pressure algometry 4 h after dehorning compared with administration of a cornual nerve block alone [6]. Ingvast-Larsson et al. described the pharmacokinetics of meloxicam in goats and observed fewer signs of distress in treated kids compared with controls after dehorning [7]. These findings indicate that administration of meloxicam at 0.5 mg/kg IV or IM decreases behavioral and physiological responses linked to pain and distress associated with cautery dehorning.

In previous studies, a reduction in signs of distress following systemic meloxicam administration prior to dehorning was achieved in combination with local anesthesia provided with a cornual nerve block [4-6]. However, a survey of North-Central and North-Eastern United States dairy producers found that only 12.4% of dairy owners use local anesthetic nerve blocks and only 1.8% provide systemic analgesia at the time of dehorning [8]. Similarly, only 18% of Wisconsin dairy producers report using local anesthetics prior to dehorning [9]. These data are consistent with the results of the recent National Animal Health Monitoring System (NAHMS) survey that reported that only 13.8 percent of U.S operations report using analgesics or anesthetics during hot iron dehorning [1]. Administration of effective systemic analgesia at the time of dehorning instead of local anesthesia may be attractive to dairy producers since cornual nerve blocks delay cattle processing because these require 10 – 15 minutes to take effect [10].

The effects of meloxicam administration without local anesthesia on post-surgical behavior and performance in older calves (> 16 weeks of age) have not been described. In addition, the compartmental pharmacokinetics of meloxicam administered intravenously to calves subjected to dehorning procedures has not been reported. If meloxicam administration alone mitigates pain and distress and is associated with quantifiable performance benefits when administered prior to dehorning, this would provide producers and veterinarians with a practical and cost-effective way to reduce pain and distress after dehorning. The primary objectives of this trial were to (1) describe the compartmental pharmacokinetics of meloxicam in calves after IV administration at 0.5 mg/kg and (2) to determine the effect of meloxicam on cortisol response, substance P (SP) concentrations, heart rate (HR), activity and weight gain in calves after scoop dehorning and thermocautery without local anesthesia.

## Results

No calves were determined to require rescue analgesia during the course of the study.

### Meloxicam pharmacokinetics

**Figure 1:**
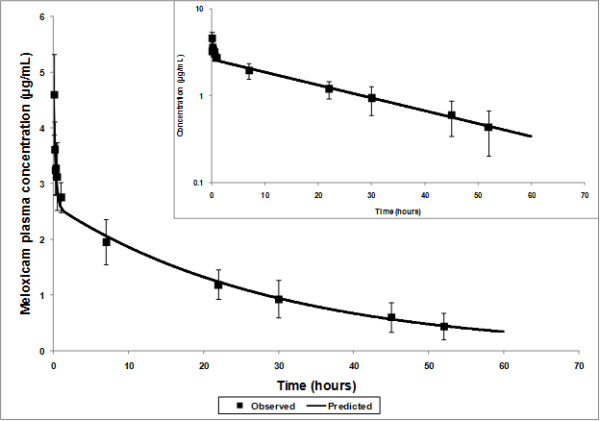
Average predicted time-concentration curve compared to observed data (± standard deviation; n = 6 calves) after administration of meloxicam at 0.5 mg/kg IV. Linear and semi-log scale (inset) plots.

The average observed and predicted plasma time-concentration curve of meloxicam following IV administration at 0.5 mg/kg bodyweight in calves is presented in Figure 1. The data fit a 2-compartment model characterized by rapid distribution of meloxicam from the central to the peripheral compartment followed by a slower decline in mean plasma meloxicam concentrations governed by metabolism and excretion processes. The mean pharmacokinetic parameters calculated after fitting this model to the data are summarized in Table 1.

**Table 1:** Mean plasma pharmacokinetic parameters for meloxicam after IV administration at 0.5 mg/kg bodyweight determined using a two- compartment model

**Parameter**	**Units**	**Mean**	**Standard error**
V_c_	mL/kg	94.88	9.04
V_2_	mL/kg	99.07	7.85
V_ss_	mL/kg	193.94	10.34
CL	mL/h/kg	6.64	0.76
CLD_2_	mL/h/kg	225.18	47.43
T_½α_	hr	0.22	0.087
T_½β_	hr	21.86	3.03
AUC	hr*ug/mL	81.02	10.58
MRT	hr	31.24	4.37
K_10_	1/h	0.075	0.012
K_12_	1/h	2.70	0.78
K_21_	1/h	2.20	0.39

V_c,_ - volume of the central compartment; V_2_ - volume of the peripheral compartment; V_ss_- apparent volume of distribution at steady-state; CL - total body clearance; CLD_2:_ - distributional clearance from the central compartment to the peripheral compartment; T_½α_ and T_½β_ half-lives for the distribution phases; AUC- area under the time-concentration curve; MRT- mean residence time of the drug in the body; k_12_ and k_21_ - μ-rate constants for the drug’s movement between the central and peripheral compartments; and k_10_ - elimination rate constant.

### Cortisol

**Figure 2:**
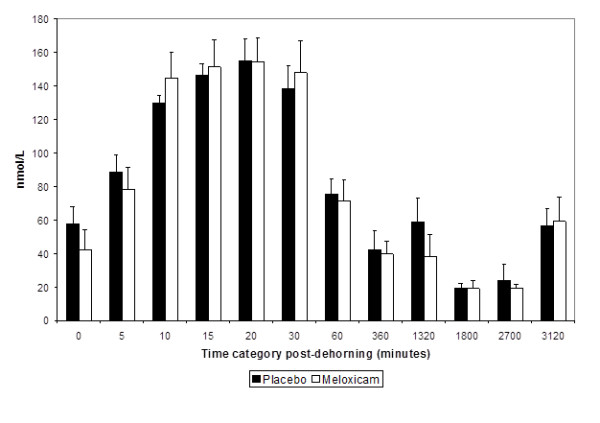
** Mean (± SEM) serum cortisol concentrations (nmol/L) in calves receiving 0.5 mg/kg meloxicam or placebo IV immediately (< 30 s) prior to dehorning. **There were no significant differences between treatments at any timepoints.

Mean serum cortisol concentrations over time for the meloxicam and placebo treated groups are presented in Figure 2. There were no significant differences in cortisol concentrations noted between treatment groups at any time point. Mean cortisol concentrations were 57.62 ± 11.62 nmol/L in the control group and 42.10 ± 11.62 nmol/L in the meloxicam treated group (P= 0.35). Cortisol concentrations in both treatment groups were significantly higher at 10, 15, 20 and 30 minutes after dehorning compared the other time points (P< 0.01). However, there was no evidence of a time by treatment interaction (P=0.85). Mean non-compartmental analysis parameters for cortisol are summarized in Table 2. There was no significant difference between Cmax, Tmax and AUEC between treatment groups (P>0.05). There was also no association between the log transformed percent change in cortisol concentrations and the corresponding plasma meloxicam concentrations at each timepoint (P=0.45).

**Table 2:** Mean peak serum cortisol concentrations (Cmax), time to peak concentration (Tmax) and area under the time-effect curve (AUEC) for cortisol determined using non-compartmental analysis

**Cortisol parameters**	**Control**	**Meloxicam**	**P value**
Tmax (min)	15.83 ± 4.92	19.17 ± 9.17	0.68
Cmax (nmol/L)	159.17 ± 11.032	165.00 ± 14.84	0.76
AUEC minute●nmol/L	133,584 ± 18,039	122,961 ± 20,441	0.70

*Cmax* Peak cortisol concentrations.

*Tmax* time to peak cortisol concentration.

*AUEC* area under the time-effect curve for cortisol determined using non-compartmental analysis.

### Substance P (SP)

**Figure 3:**
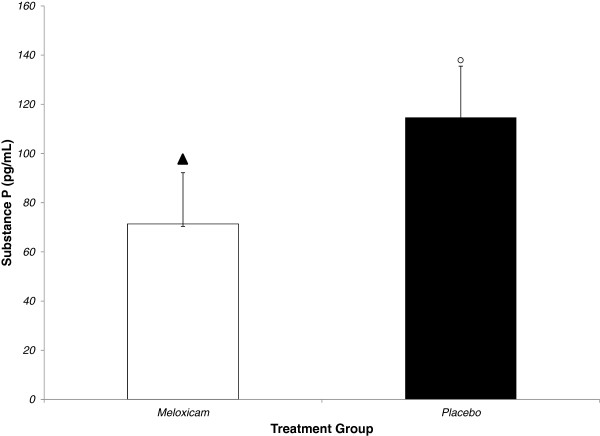
** Mean (± SEM) plasma Substance P concentrations (nmol/L) in calves receiving 0.5 mg/kg meloxicam or placebo IV immediately (< 30 s) prior to dehorning. **Columns not connected by a symbol of the same shape and color are significantly different (P < 0.05).

**Figure 4:**
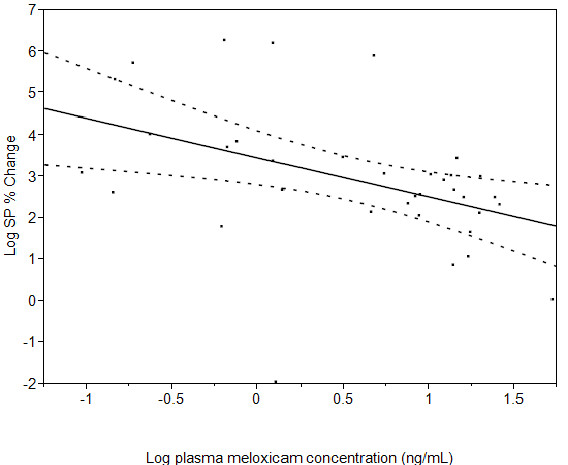
** Linear regression fit (solid line) and 95% confidence interval (dotted line) of log transformed plasma substance P (SP) percent change from baseline concentrations compared with log meloxicam concentrations. **There was a negative correlation between log meloxicam concentrations and% change in SP (P = 0.008) described by the equation Log SP% Change = 3.4475533 - 0.9440988*Log Meloxicam.

Mean (± SEM) SP concentrations were 114.70 ± 20.84 pg/mL in the control calves and 71.36 ± 20.84 pg/mL in the meloxicam treated calves (P=0.038) (Figure 3). There was no evidence of an effect of time (P=0.29) or a time by treatment interaction (P=0.16) on log-transformed plasma SP concentrations. The back transformed estimate of the difference between average SP concentration in meloxicam and placebo-treated calves was 0.50 (95% Confidence interval: 0.26 to 0.96). Therefore, the plasma SP concentration is estimated to be 0.5 times less in the presence of meloxicam treatment than in the absence of treatment in calves after dehorning (95% Confidence interval: 0.26 to 0.96 times). Furthermore, there was an inverse relationship between log-transformed meloxicam concentrations and log-transformed SP percent change from baseline (P=0.008) with the regression curve described by the equation Log SP% Change = 3.4475533 - 0.9440988*Log Meloxicam (Figure 4).

### Activity and behavior

**Figure 5:**
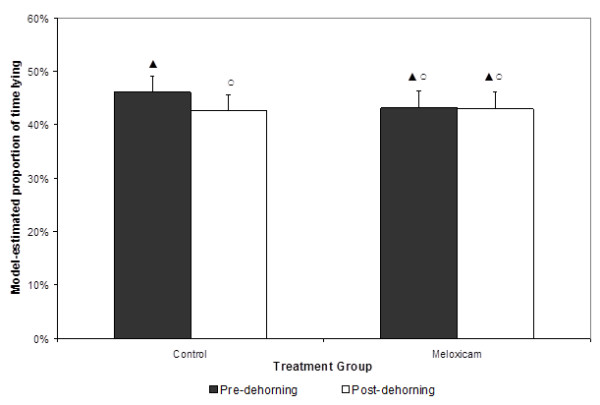
** Model estimated proportion of time calves spent lying by treatment group and time relative to dehorning (pre = 48 h, post = 168 h). **Model included random effects for calf identification and trial replicate. Columns not connected by a symbol of the same shape and color are significantly different (P < 0.05).

Data from one calf (calf number 7, meloxicam group) was not available for analysis due to accelerometer malfunction resulting in loss of data. The effect of dehorning on lying behavior was modified by meloxicam administration, as evidenced by the significant (p< 0.01) interaction between time relative to dehorning (pre vs. post) and treatment group (meloxicam vs. control). Calves in the control group spent a lower proportion (42.7%) of time lying post-dehorning compared to pre-dehorning (46.1%); however, there were no significant differences (p=0.40) in the proportion of time the meloxicam calves spent lying pre- or post-dehorning (43.1%, 43.0%, respectively) (Figure 5).

### Heart rate (HR)

**Figure 6:**
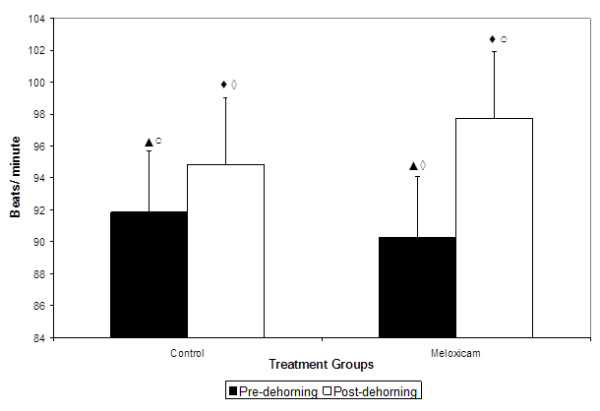
** Mean (± SEM) heart rate (beats/min) in calves over 48 h before and after receiving 0.5 mg/kg meloxicam or placebo IV immediately (< 30 s) prior to dehorning. **Columns not connected by a symbol of the same shape and color are significantly different (P < 0.05).

After periods of missing data were removed there were 156,398 data points available for analysis. The comparison of mean heart rate in the period before and after dehorning in meloxicam and placebo treated calves is presented in Figure 6. The mean HR was not significantly different between calves assigned to the control group (91.85 ± 3.82 beats/minute) and the meloxicam treated group (90.27 ± 4.19 beats/minute) prior to dehorning (p= 0.79). However, after dehorning, the mean HR in calves in the placebo treated control group increasing to 94.83 ± 3.82 beats/minute (p < 0.0001) and in the meloxicam treated calves increased to 97.72 ± 4.19 beats/minute (p< 0.0001). There was however no difference in overall HR between the two treatment groups during the entire post-dehorning period (p=0.62).

**Figure 7:**
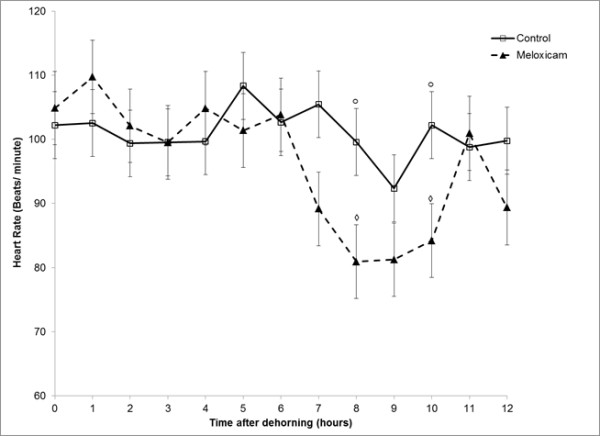
** Mean (± SEM) heart rate (beats/min) in calves collected every 15 s over 12 h after receiving 0.5 mg/kg meloxicam (▴) or placebo (□) IV immediately (< 30 s) prior to dehorning. **Data points not connected by a symbol of the same shape are significantly different (Pthinsp;< 0.05).

In order to address issues with model stability and convergence when examining hourly changes in HR after treatment, data were truncated to 20,000 data points collected at 15 sec intervals over 12 h after dehorning (Figure 7). This allowed evaluation of the effect of meloxicam treatment on hourly HR during the period immediately following dehorning. There wasevidence of an effect of time (P <0.0001) and a time by treatment interaction (P <0.0001) on HR over this period. Mean HR was significantly elevated over the first 6 hours after dehorning (Range: 99.85 – 106.13 beats/minute) (P < 0.05) compared with the heart rate from 7 to 12 h after dehorning (Range: 86.79 to 97.30 beats/minute). The difference in mean HR in calves treated with meloxicam compared to placebo-treated calves approached significance at 7h after dehorning (P=0.0639) and was significantly lower at 8 h (P=0.034) and 10 h (P=0.045) after dehorning.

### Average daily gain (ADG) in bodyweight

**Figure 8:**
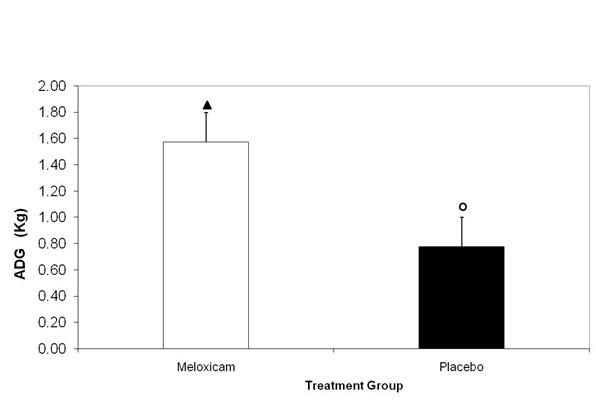
** Mean Average Daily Gain (ADG) (Kg) (+/− SEM) 10 days post-dehorning after placebo or meloxicam administration at 0.5 mg/kg IV prior to dehorning. **Columns not connected by a symbol of the same shape and color are significantly different (P < 0.05).

During the 19 days preceding the current study, there was no significant difference (P= 0.41) in mean ADG in body weight (± SD) for calves in the meloxicam group (0.86 ± 0.19 kg/calf/day) and the control group (1.02 ± 0.41 kg/calf/day). The mean ADG in calves in the placebo or meloxicam treated groups 10 days after dehorning is presented in Figure 8. Calves receiving meloxicam prior to dehorning gained an average of 1.05 ± 0.13 kg/day over 10 days post dehorning. This was significantly greater than the mean average daily gain in bodyweight of 0.40 ± 0.25 kg/day in the control group over the same period (p=0.0418).

## Discussion

The primary objectives of this trial were to describe the compartmental pharmacokinetics of meloxicam in calves after IV administration at 0.5 mg/kg and to determine the analgesic effect of meloxicam after scoop dehorning and thermocautery without local anesthesia. Surgery-induced pain and central sensitization consist of two phases: an immediate incisional phase and a prolonged inflammatory phase that arises primarily due to tissue damage [11]. Demonstrating the adequacy of preemptive analgesia has two basic requirements [11]. The first is to demonstrate the direct pharmacological effect of the analgesic. This was accomplished in the present study by comparing differences in acute biomarkers of pain and distress including substance P, cortisol response and heart rate between treated and control subjects. The second requirement is to demonstrate the extension of the antinociceptive effect into the postoperative period when pain due to inflammation becomes the dominant process [11]. In practical terms, two approaches have been used to demonstrate the efficacy of preemptive analgesic regimens. The first is to demonstrate a reduction in pain intensity beyond the presence of the drug in the biophase in studies involving treated and untreated control subjects. The second approach is to demonstrate that a treatment applied before surgery is more effective than the treatment applied at the end of surgery [11]. In the present study, we used pharmacokinetic analysis to determine the presence of meloxicam in the biophase and cortisol, SP and heart rate analysis to determine the direct pharmacological effect of meloxicam in treated and untreated calves. Continuous, telemetric assessment of posture and activity over 96 hours after dehorning and weight gain over 10 days was used to determine if meloxicam effects extended into the post-operative period.

In the present study, plasma meloxicam concentrations were still quantifiable in the last sample, at 52 hours post-injection, and showed a bi-exponential decay following administration. The initial steep decline in plasma concentrations was likely due to the rapid distribution of the drug from the central to the peripheral compartment. This was followed by a slower decline in plasma concentrations associated with drug metabolism and excretion. Although exceptions due to species and drug compound exist, NSAIDs in general tend to be highly protein-bound in the plasma which limits distribution into the tissue, leading to low volume of the central compartment and low volume of distribution [12] as was seen in the current study. The extended half-life of meloxicam in cattle is likely due to a low total body clearance representing mostly hepatic clearance since high levels of protein binding tend to limit glomerular filtration of drug compounds.

The clinical implication of the slow elimination of meloxicam from the body is that infrequent drug administration (once every few days) may be sufficient to mitigate pain effects due to post-surgical inflammation in calves. It was recently reported that generic meloxicam tablets have 100% bioavailability following oral administration in ruminant calves suggesting that this may provide a practical and cost effective alternative to IV administration in those animals [13]. The pharmacokinetic profile of meloxicam described in the current report along with the associated effects on behavior and performance suggest that administration immediately prior to dehorning may have effects for several days post-dehorning. Given that the plasma half-life of meloxicam is longer than previously reported for ketoprofen (0.42 h) [14], salicylate (0.5 h) [15] and flunixin (4- 8 h) [16], these results suggest that meloxicam may have an extended duration of activity compared with other NSAIDs currently available in the U.S. However, further research is needed to determine if the effect of these analgesics is directly related to plasma drug concentrations, and if so, to determine the effective range. Although the literature is deficient in studies with cattle, the effective plasma concentration (EC50) of meloxicam is estimated to be 0.73 μg/mL in the horse [17] and 0.36 μg/mL in the dog [18]. If cattle respond to meloxicam as horses and dogs, the effective plasma drug concentration would be maintained for several days following IV administration of meloxicam at 0.5mg/kg.

Cortisol, substance P and heart rate analysis was used as an indicator of the direct pharmacological effect of meloxicam on pain and distress associated with dehorning in treated and untreated calves. It has been reported that plasma cortisol concentrations reach a peak within 30 minutes after dehorning after which levels decrease to a plateau concentration that persists for 5 – 6 hours [19,20]. The results presented here are consistent with previous reports that demonstrate a significant increase in plasma cortisol concentration after dehorning [4,5,21]. However, in contrast with the findings of Heinrich and others [4], the present study failed to demonstrate that calves receiving meloxicam prior to dehorning had lower cortisol concentrations compared with untreated controls. This may be due to differences in the blood collection schedule that was designed to minimize the effect of animal handling on behavioral assessment. Furthermore, the present study enrolled older calves (<3mo vs. >4 mo) and necessarily employed a different method of dehorning (cautery disbudding vs. amputation). Since the horn bud attaches to the skull of calves at approximately 2 months of age, then gradually develops a diverticulum communicating with the frontal sinus, removal of the horn tissue is generally considered more invasive and stressful in older animals resulting in higher cortisol concentrations [22].

The most likely explanation for the differences in cortisol response is that previous reports frequently combined NSAID administration with local anesthesia. Local anesthetics mitigate acute incisional pain by blocking voltage-gated sodium channels in the nerves preventing the generation and propagation of nerve impulses or action potentials [10]. In the bovine, this effect lasts approximately 2 – 3 h after dehorning with the provision of a lidocaine block of the cornual nerve [10,20]. Lidocaine local anesthesia combined with systemic ketoprofen administration prior to dehorning significantly attenuates the plasma cortisol response compared with the effect of the agents administered separately [19,20,23]. The reason why we chose not to administer local anesthesia in the present study was because surveys suggest that less that 20% of U.S. dairy producers currently use local anesthetics prior to dehorning and our goal was to look at the effect of an NSAID without local anesthesia [1,8,9]. The absence of an effect of meloxicam on acute cortisol response suggests that provision of systemic analgesia alone without local anesthesia is inadequate in providing adequate preemptive analgesia using the definition provided by Kissin, 2000 [11].

Substance P is an 11-amino acid prototypic neuropeptide that regulates the excitability of dorsal horn nociceptive neurons and is expressed in areas of the neuroaxis involved in the integration of pain, stress, and anxiety [24]. It has been reported that plasma SP concentrations are significantly higher in beef calves after castration compared with uncastrated controls [25]. In the present study, mean plasma substance P concentration was estimated to be reduced by 50% (95% Confidence interval: 26 to 96%) in calves that received meloxicam prior to dehorning compared with placebo-treated controls. Furthermore, we observed that increases in plasma substance P concentrations from baseline corresponded with lower log plasma meloxicam concentrations. These findings support the hypothesis that meloxicam treatment mitigates SP release and therefore potentially reduced pain perception in calves after dehorning without local anesthesia. To our knowledge this is the first study that has demonstrated a relationship between substance P concentrations and analgesic drug concentrations after dehorning. This suggests that SP measurement may have potential to be used as a biomarker for determining analgesic drug efficacy in calves subjected to painful procedures. Furthermore, the absence of an effect of meloxicam treatment on serum cortisol concentrations suggests that simultaneous determination of plasma SP and cortisol concentrations during painful procedures may assist in differentiating between acute stress associated with handling and distress associated with nociception as previously described [25].

Normal HR in unstressed cattle range from 70 to 90 bpm [26] but mean HR has been shown to increase by 30 to 40 bpm over baseline levels by stressful events such as branding, electric shock and handling prior to dehorning and castration [21,27,28]. Schwartzkopf-Genswein and others reported that HR in dehorned calves was significantly higher that control animals for 120 minutes after the procedure [21]. Similarly, Grondahl-Nielsen et al. (1999) observed that HR was elevated for 3.5 h in dehorned calves receiving no anesthetic or analgesic compared with calves that were only sham dehorned [29]. Likewise, Stewart et al, 2009 observed that HR was raised above baseline for 3 h after dehorning without local anesthesia [5]. Heinrich et al (2009) observed a greater increase in HR, measured by thoracic auscultation at 7 time points over 24 h, in placebo-treated calves compared with calves that received 0.5 mg/kg meloxicam IM combined with corneal nerve block at 10 minutes prior to dehorning [4]. In the present study, baseline HR was higher than previously reported and were maintained above 100 bpm for 6 hours after dehorning in both treatment groups. This difference could be attributed to the use of older calves (16 – 20 wks vs. < 6 wks) than what had been used in previous experiments. In our study we observed a significant decrease in mean HR in meloxicam-treated calves at 8 and 10 hours after dehorning. These results support the conclusions of Heinrich and others (2009) [4] that meloxicam reduces the stress response after dehorning as reflected by in changes in heart rate even in the absence of local anesthetic administration at the time of dehorning.

Comparison between calf behavior before and after dehorning and assessment of weight gain over 10 days post-dehorning was used to determine if meloxicam effects extended into the post-operative period. It was recently reported that calves that received oral meloxicam at 1 mg/kg spent more time lying down over 4 days compared with placebo-treated control calves [30]. The amount of time cattle exhibit specific behaviors is commonly used to indicate comfort and/or clinical illness [31-34]. An increase or decrease in lying behavior, however, does not expressly indicate pain or comfort and must be interpreted within the context of what is normal for a particular animal. Cattle in pain due to lameness have been observed to lie more [35] whereas cattle in pain due to dehorning and castration have been observed to lie less than nonpainful controls [6,30,36,37]. Furthermore, lying behavior among individual cattle within a herd has been observed to vary more than lying behavior between herds [34]. Therefore, within-animal comparisons, such as implicitly occurs with an analysis such as in the current study comparing an individual’s control period (pre-procedure) and treatment period (post-procedure) behavior, are likely to be more sensitive than between-animal comparisons in detecting changes due to a particular treatment. Since meloxicam-treated calves were intermingled equally with placebo-treated calves, the difference noted between groups in the pre-procedure period is likely due to individual variation in the normal amount of time spent lying.

It has been previously reported that calves that received meloxicam at 0.5 mg/kg IM combined with corneal nerve blocks were less active than controls during the first 5 h following dehorning compared with placebo-treated controls [6]. In the present study, calves receiving meloxicam without local anesthesia demonstrated no significant difference in lying activity before and after dehorning. In contrast, control calves spent less time lying after dehorning, and although the percentage difference was numerically small (3.4%), this difference was significant due to the associated small standard error. This is similar to the results reported by Theurer and others (2012) that observed differences in lying behavior for 5 days after dehorning in calves that received oral meloxicam at 1 mg/kg [30]. When considering a 3.4% difference over a 24 hour period, control calves stood an average of 49 minutes more per day after dehorning than before. In meloxicam calves, the absence of an effect of dehorning on lying behavior may have been due to the analgesic activity of the drug. These results suggest that meloxicam mitigates behavioral effects of dehorning even in the absence of local anesthetic administration.

Studies reporting a difference in weight gain between analgesic-treated and unmedicated calves after dehorning are deficient in the published literature. Previously, Faulkner and Weary (2000) demonstrated that calves receiving ketoprofen prior to dehorning tended to gain more weight during the 24 h after dehorning than untreated calves [38]. However, during the subsequent 24 hour period, weight gains were similar between the two groups. Administration of the NSAID, sodium salicylate in drinking water for 2 d after dehorning and castration was found to increase ADG over 13 d [39]. Over the 10 day duration of the present study, calves receiving meloxicam gained significantly more weight than those in the control group, with a mean difference of 0.65 ± 0.28 kg/day (p=0.0418). This finding supports the hypothesis that extended exposure to a non-steroidal anti-inflammatory drug (NSAID) may maintain growth and performance after castration.

Although studies reporting a performance benefit after NSAID administration 2- 3 weeks following processing and castration are deficient in the published literature, meloxicam administration has been associated with improved average daily gain in calves suffering from clinical bovine respiratory disease [40]. Therefore, in order to definitively attribute the observed difference in ADG to the effect of meloxicam on pain and inflammation associated only with dehorning, future studies should also include additional meloxicam treated and untreated control calves that are processed but not dehorned.

The biological explanation for the improved ADG observed in this study was not investigated. Meloxicam treated and untreated control calves were co-mingled in pens for the duration of the study precluding assessment of individual animal feed intake or feed efficiency. Furthermore, even though the accelerometer analysis revealed significant differences in the pre and post-dehorning behavior of control calves, this technology is currently not sufficiently sensitive to characterize subtle differences in feeding and walking behavior that could contribute to performance differences. However, Theurer and others [30] recently reported that calves that received oral meloxicam prior to dehorning spent more time at the grain bunk and less time at the hay feeder compared to the control group which could explain the difference in weight gain observed in the present study. Another possible contributory factor to the performance difference is that increased activity of nociceptors increases sympathetic tone and adrenal secretory activity which may inhibit gastric centers causing decreased rumen motility [41]. Mellor and others (2002) reported a significant increase in plasma adrenaline and noradrenaline concentration for 5 and 50 minutes respectively after scoop dehorning in 10 week old calves [42]. Adrenergic influences on reticuloruminal motility comprise depression of the gastric centers resulting in inhibition of intrinsic and extrinsic rumen motility [43]. The duration of these effects and how these relate to plasma catecholamine concentrations has not been reported. Future studies examining individual or pen level feed intake and feed efficiency and the association with rumen motility after dehorning may help explain the difference in ADG observed in this report.

## Conclusion

The results of this trial support the conclusions of previous reports that observed a significant effect of meloxicam on behavior and heart rate changes after dehorning with local anesthesia [4,6,30]. However, we failed to detect an effect of meloxicam administered without local anesthesia on serum cortisol concentrations. This suggests that systemic administration of an NSAID does not adequately mitigate acute signs of distress associated with dehorning. This study contributes to the body of literature by demonstrating for the first time that meloxicam administration significantly reduces plasma substance P concentrations and that an inverse relationship exists between meloxicam concentrations and changes in circulating SP concentration after dehorning. To our knowledge, this is also the first published report that observed a significant effect of meloxicam administration on plasma substance P concentrations and weight gain after dehorning. These results have implications for developing pain mitigation strategies involving NSAIDs in calves at dehorning with respect to addressing both animal performance and welfare concerns.

## Methods

All experimental procedures in this study were approved by the Kansas State University (KS) Institutional Animal Care and Use Committee (IACUC) under the supervision of the University Veterinarian (Protocol #2694). Since a placebo-treated, dehorned control group was enrolled in the study, calves were assessed hourly for behavioral signs of excessive pain over a period of 10 hours after surgery. This was followed by twice daily monitoring for 7 days. Calves demonstrating postural changes, prolonged recumbency, anorexia and depression were scheduled to receive rescue analgesia with flunixin meglumine at 2.2 mg/kg IV, BID.

### Animals

Twelve Holstein calves approximately 16-20 wks of age and weighing between 140-205 Kg were acquired from a commercial dairy located in South-West Kansas. Upon arrival, the calves were given an eight-way clostridial vaccine (Covexin 8, Schering Plough), a single SQ injection of tulathromycin (Draxxin, Pfizer) at 2.5 mg/kg bodyweight, and doramectin (Dectomax Pour-on, Pfizer) administered topically at 500 μg/kg bodyweight. Amprolium (Corid, Merial) was added to the drinking water to provide 10 mg/kg PO for 5 days. Calves were maintained in study housing facilities for 19 days prior to study commencement.

### Randomization and group assignment

Calves were blocked in pairs according to their weights determined approximately 14 days prior to study commencement. Calves were ranked by ascending weight in kilograms and assigned a computer-generated random number (Microsoft Excel 2007, Microsoft Corporation). In each pair, the calf with the highest random number was assigned to the meloxicam-treated group, while the calf with the lowest random number was designated as a placebo-treated control (n=6 calves/group).

### Housing and husbandry

Calves were housed in groups of 6 animals (n=3 steers from each treatment group in each pen) in a dry lot confinement facility at KSU Animal Resource Facility for the duration of the study. Housing consisted of an outdoor concrete pad (9.75m x 18.29m) with a partial roof on straw bedding. During the adaptation period, each calf was tied with a rope halter to the pole fence within their pen for at least 10 minutes/day. Calves had free access to water and brome hay for the entire housing period. A balanced feedlot receiving ration composed of cracked corn, wheat middlings, oats, soymeal and a protein/vitamin/mineral supplement (Table 3) was fed at 3.6 kg/head/day.

**Table 3:** Ration nutrient composition on an as fed and dry matter basis

**NUTRIENT**	**COMPOSITION**	
	**AS FED**	**DRY**
NEm Megcal/CWT.	83.10	95.23
NEg Megcal/CWT.	54.62	62.59
TDN%	76.46	87.62
Fat	4.61	5.29
Crude Fiber	4.18	4.79
ADF	5.61	6.43
NDF	12.84	14.71
eNDF	26.56	30.43
Crude Protein%	13.65	15.64
Potassium%	0.60	0.69
Calcium%	0.33	0.38
Phosphorus%	0.35	0.40
Magnesium%	0.14	0.16
Sulfur%	0.24	0.28
Cobalt ppm	0.06	0.07
Copper ppm	5.94	6.8
Iron ppm	50.64	58.0
Manganese ppm	20.75	23.8
Selenium ppm	0.14	0.17
Zinc ppm	309.55	354.7

NEm Megcal/CWT –Megcal of Net Energy for Maintenance (NEm) available per 100 lb (CWT) of feed; NEm Megcal/CWT – Megcal of Net Energy for Maintenance (NEm) available per 100 lb of feed; TDN - Total Digestible Nutrients; ADF - Acid Detergent Fiber; NDF – Neutral Detergent Fiber; eNDF – Effective NDF; ppm – parts per million.

### Catheterization and acclimatization

Approximately 48 hours prior to study commencement, calves were restrained for jugular catheter placement under local anesthesia. All study animals were fit with two catheters, one catheter was designated for drug or placebo administration and the other for blood sample collection. Catheter patency was maintained using heparin saline flush containing 3 USP units heparin sodium/ml saline (Heparin Sodium Injection, Baxter Healthcare). Catheters were removed immediately following drug administration or final blood collection.

Following catheter placement, calves were restrained twice daily using a rope halter to simulate study sampling procedures. Furthermore, calves were run through the chute handling facilities once daily and manipulated in the same manner as the proposed study procedures. Cattle were also fitted with commercially manufactured 3-dimensional accelerometers (GP1 SENSR, Reference LLC) at described in previous studies [44]. The accelerometers were left on the calves until study completion, approximately 7 days after placement.

### Treatment administration

Calves were subjected to either meloxicam or placebo treatment as outlined below (n=6 steers/treatment). Doses were calculated based on individual animal bodyweight determined 14 hours prior to study commencement. The IV dose was rounded to the nearest 0.5 ml and administered using a 20 mL syringe. Meloxicam or the placebo was administered immediately (<30 seconds) prior to commencement of the dehorning procedure.

1) Intravenous (IV) injection of 0.5 mg/kg of meloxicam (Metacam 5 mg/ml solution for injection (NADA 141-219), Boehringer Ingelheim Vetmedica, Inc; Lot # 118ZN12) was administered as a single bolus in the jugular vein using a designated catheter. The catheter was flushed with 5 mL of heparin-saline and removed immediately after administration.

2) Intravenous sodium chloride injection (0.9% Sodium Chloride Injection USP, Baxter Healthcare Corp) was administered at a volume based on a presumed dose of 0.5 mg/kg meloxicam injection.

Observers and analytical chemists in the study were masked to treatment group allocation.

### Dehorning

Prior to dehorning, all calves were restrained in a chute with a head gate and a rope halter. The horn was removed using a Barnes dehorning instrument (Stone Manufacturing & Supply Company). Briefly, the opposing blades of the instrument were aligned with the base of the horns at the skin-horn junction. The handles of the instrument were then closed slowly to ensure proper placement of the instrument. Once optimal positioning was achieved, the handles were spread quickly apart to engage the blades and cut off the horn. Hemostasis was achieved through thermocautery using a pre-heated electric dehorning iron (Stone Manufacturing & Supply Company). All dehorning procedures were performed by a single experienced veterinarian (BR). After dehorning, calves remained standing but unrestrained in the chute for 20 minutes to facilitate intensive blood sampling. Subsequent samples were collected in housing pens with calves periodically restrained using a rope halter.

### Blood sample collection

Eighteen milliliters of whole blood for cortisol, substance P (SP) and meloxicam determination was collected into syringes using the pre-placed jugular catheter immediately prior to drug or placebo administration, and at 5, 10, 15, 20, 30, 60 minutes and again at 6, 22, 30, 45 and 52 hours thereafter. Blood was immediately transferred to 6 ml serum and lithium heparin vacutainer tubes (BD Diagnostics) for cortisol and drug determination respectively. Blood for SP determination was collected in 6 ml EDTA tubes containing the serine protease inhibitor, aprotonin at 500 KIU/mL of blood. The vacutainer tubes were stored on ice for no more than 60 minutes pending sample processing. Thereafter, blood samples were centrifuged at 1,600 g for 15 minutes at 4°C. Serum and plasma were pipetted from their respective tubes and placed in cryovials for storage at -80°C prior to sample analysis. All samples were analyzed within 60 days of sample collection.

### Cortisol determination

Serum cortisol concentrations were determined using a solid-phase competitive chemiluminescent enzyme immunoassay and an automated analyzer system (Immulite 1000 Cortisol, Siemens Medical Solutions Diagnostics) as previously described using an assay validated in bovine plasma [15,45]. A sample volume of 100 μL was used in each assay well. The reported calibration range for the assay is 28 to 1,380 nmol/L with an analytical sensitivity of 5.5 nmol/L.

### Substance P determination

Plasma substance P concentrations were determined in duplicate using a validated method as previously described [25]. Briefly, Substance P was extracted from plasma by acidifying with acetic acid and fractionating with reverse-phase solid-phase extraction columns. The peptide was eluted from the column using an organic-aqueous solvent mixture and concentrated by drying under nitrogen. The dried extract was reconstituted and analyzed according to the manufacturer’s instructions in the Substance P ELISA kit (Assay designs, Ann Arbor, MI). The coefficient of variation between triplicate bovine samples at each fortified SP concentration was < 30%. The linear regression line fit between the three points at each of three control concentrations had a correlation coefficient of 0.99.

### Accelerometers

Activity data was collected for 48 and 168 hours pre- and post-dehorning, respectively. Accelerometers sampled at 100Hz and summarized values for the selected variables every 5 seconds. Five variables were recorded by the accelerometers for each 5-second interval: average acceleration in each of the three axes (X, Y, and Z), the combined average magnitude for all three axes, and the maximum combined vector magnitude. At trial completion, data were imported into commercial data mining software (Insightful Miner, Insightful Corporation), and a previously validated decision tree [30,36,44] was used to classify the behavior as lying, standing, or walking for each 5 second interval.

### Heart rate determination

Heart rate data were recorded for 48 hours before and after dehorning for each calf. Heart rate was recorded and analyzed using a commercially available heart rate monitor and software (RS800 and Polar Pro Trainer Equine Edition, Polar Electro, Inc, Lake Success, NY) as previously described [45]. The heart rate monitor consisted of a transmitter placed over the heart in the left foreflank attached to a girth strap placed around the heart girth of the calves, and a wrist unit attached to the elastic strap which received and recorded the signal from the transmitter. Appropriate conductance for the electrodes on the strap, one positioned on the sternum and one over the right scapula, was facilitated by use of ultrasound gel (Medline Industries Inc. Mundeline, Il). The transmitter measured the electric signal (ECG) of the heart every 15 seconds. Prior to study commencement, the heart rate wrist unit time was synchronized with the stopwatches used for all other sample collection. The corresponding heart rate within 15 seconds of each time point was used for analysis.

### Average daily weight gain (ADG)

Calves were individually weighed on arrival, approximately 14 hours prior to dehorning and 10 days post-dehorning using a commercial livestock scale (For-Most Livestock Equipment). Food and water were not withheld prior to weighing. Average daily gain (ADG) was calculated by subtracting the arrival from the pre-dehorning weight and the pre-dehorning weight from the post-dehorning weight and dividing this by the number of days that passed between weigh dates.

### Plasma meloxicam determination

Plasma concentrations of meloxicam (*m/z* 352.09→114.90) were determined with high-pressure liquid chromatography (Shimadzu Prominence, Shimadzu Scientific Instruments) and mass spectrometry (API 2000, Applied Biosystems) as previously described [13,46]. With a limit of quantification of 0.025 μg/mL, the standard curve was linear from 0.025 μg/mL to 10 μg/mL and was accepted if the correlation coefficient exceeded 0.99 and predicted values were within 15% of the actual values. The accuracy of the assay was 103 ± 7% of the actual value and the coefficient of variation was 7% determined on replicates of 5 each at 0.025, 0.5, and 5 μg/mL.

### Pharmacokinetic analysis

Compartmental pharmacokinetic analysis of the meloxicam time concentration data was performed using a commercially available software program (WinNonlin, Pharsight Corporation) as previously described [15,47]. Model selection was conducted using visual inspection of predicted versus observed data plots and two measures of goodness of fit (Aikaike Information Criterion and Schwarz Bayesian Criterion).

### Data analysis and statistics

Hypothesis tests were conducted using JMP 5.1.2 analytical software (SAS Institute, INC) unless otherwise specified [48]. For statistical analysis, the calf was considered the experimental unit. The mean ± standard error of the means (SEM) and the mean difference where appropriate were calculated for each outcome variable at each time point. Statistical significance was designated *a priori* at p<0.05. Model fit was assessed by evaluating the form of marginal studentized residuals versus fitted values plot. The model was determined appropriate if the mean of the residuals versus fitted values plot was centered on zero.

For repeated measures data (heart rate, substance P and cortisol concentrations at each time point), a random effects-mixed model was constructed with treatment, time and the interaction between time and treatment designated as fixed effects. In this model, animal nested in treatment was designated as a random effect to account for the between subject effects. Where significant interactions were detected, comparisons between different combinations were conducted using two-sided Student t-tests. The experiment wise significance level was protected by employing the Tukey method in examining comparisons. Substance P data were not normally distributed and therefore these were log transformed prior to statistical analysis. For heart rate data, the statistical model failed to converge due to the large number of data points relative to the number of animals on trial and periods of missing data especially after 12 h post-dehorning. Therefore, an initial analysis was conducted on the cumulative data collected in the periods before and after dehorning followed by a detailed analysis of the hourly data over 12 h post-dehorning.

Accelerometer data were imported into a commercial data mining software (Insightful Miner, Insightful Corporation, Seattle, WA), and a previously validated decision tree [36,44] was used to classify the behavior as lying, standing, or walking for each 5 second interval. Following classification, data were aggregated on an hourly basis by the summing of the counts of 5-second intervals spent in each behavior. Hours with known periods of human intervention (feeding, sample collection, animal processing, and treatment administration) were removed equally from calves in both treatment groups. The remaining hourly behavioral data from both replicates of the trial were imported into a statistical program (SAS 9.1, SAS Institute, Cary, NC) for analysis. The proportion of time calves spent in each activity was modeled using logistic regression (PROC GLIMMIX) to evaluate potential associations between lying behavior and time relative to dehorning (pre- or post-), treatment (Meloxicam or control), and the interaction between these variables. Random effects were included in the model to account for a lack of independence in each sampling due to multiple calves housed within the same pen and repeated measures on individual calves. Pairwise comparisons were performed to determine statistically significant differences.

Summary measures of cortisol including peak plasma concentration (Cmax), time to peak concentration (Tmax) and area under the time-effect curve (AUEC) were determined as previously described [47] using the linear trapezoidal rule and WinNonlin software (Pharsight Corporation, Cary, NC). Analysis of variance (ANOVA) was employed to evaluate differences in single measurement, normally distributed data (ADG, Cmax, and AUEC). The Kruskal-Wallis Test was used to analyze non-parametric data (Tmax).

In order to determine the relationship between plasma meloxicam concentrations, SP and serum cortisol concentrations at the corresponding time points after dehorning, *post hoc* linear regression curves were constructed using the log-transformed percentage change from baseline. Statistical significance was designated *a priori* at p<0.05.

## Competing interests

A provisional patent application (PCT/US2011/044017) titled “Oral Administration of Meloxicam in Cattle for Improved Growth following Dehorning and Reduction of Bovine Respiratory Disease upon Castration” was filed by Kansas State University on July 14, 2011.

## Authors’ contribution

JFC conceived the study, assisted with the animal phase of the trial, conducted the statistical analysis and prepared the manuscript for publication. RAM assisted with the animal phase of the study, statistical analysis and manuscript preparation. BK conducted the meloxicam extraction and analysis. RG conducted the pharmacokinetic analysis. BR assisted with the collection and analysis of the accelerometer data. JBR conducted the dehorning procedures and BJW conducted the statistical analysis of the accelerometer data. All authors read and approved the final manuscript.
